# Need strength, perceived need support, stress symptomatology, and performance in the context of oral exams: A typological approach

**DOI:** 10.3389/fpsyg.2022.992314

**Published:** 2022-12-14

**Authors:** Linda Schürmann, Tobias Kärner, Tobias Ringeisen

**Affiliations:** ^1^Department of Developmental Psychology and Psychological Diagnostics, Institute of Psychology, University of Koblenz-Landau, Koblenz, Rhineland-Palatinate, Germany; ^2^Chair of Economic and Business Education (560A), University of Hohenheim, Stuttgart, Baden-Württemberg, Germany; ^3^Chair of Applied Psychology, Berlin School of Economics and Law, Berlin, Germany

**Keywords:** basic needs, oral exams, cortisol, stress perception, achievement emotions

## Abstract

**Introduction:**

Based on self-determination theory, we investigated whether examinees are classifiable into profiles based on basic need strength and perceived need support that differ in stress parameters and achievement in the context of a standardized oral exam.

**Methods:**

92 students reported their basic need strength before and perceived need support provided by the examiner once after the exam. Students indicated their emotions and stress perception at four measurement points and we measured their saliva cortisol concurrently, analyzing stress-related changes over time.

**Results:**

Latent class analyses revealed two higher-quality (low/high, high/high) and two lower-quality (low/low, high/low) need strength/need support classes. Physio-affective stress development was typical of exam situations. Higher-quality classes that met or exceeded the needs displayed more beneficial stress and emotion response patterns than lower-quality classes. Gain-related emotions mediated achievement in the higher-quality classes.

**Discussion:**

Need-supportive examiners can promote student well-being and achievement when they succeed in providing high need satisfaction.

## Introduction

Three basic psychological needs energize human behavior: the needs for autonomy, competence, and relatedness. The basic psychological needs are fundamental to human nature and must be satisfied for an optimal sustainment of psychological interest, development, and wellness (Ryan and Deci, 2017). The basic needs play a crucial role in student learning and performance in academic settings. When an environment supports the basic needs, for instance, when a teacher creates a learning atmosphere that the students perceive as need-supportive, the students’ resulting need satisfaction can positively affect their motivation, academic performance, and well-being (Ryan and Deci, 2017). Vice versa, a lack of teacher need support may result in unsatisfied needs, entailing negative consequences like stress and lower achievement (Reeve and Tseng, 2011; Ryan and Deci, 2020). Importantly, teachers’ need support behavior affects their students’ motivation indirectly, as it is the students’ *perception* of need support and the resulting need satisfaction that inform their motivational quality (Ryan and Deci, 2017).

The functionality of the basic psychological needs is universal and, therefore, applies to all individuals. However, the value, desire, or salience of the basic psychological needs for the individual may vary, which represents need strength (Ryan and Deci, 2017; Vansteenkiste et al., 2020). Differences in need strength might play a role in the configurations of the effects of need strength and experienced need satisfaction (Ryan et al., 2019; Vansteenkiste et al., 2020). Therefore, it can be fruitful to consider intensity patterns of both the strength and the satisfaction of the three basic psychological needs in a parallel manner, for which person-centered approaches such as latent class analyses are suitable.

Despite solid evidence for the beneficial effects of need-supportive teacher behaviors (Gilbert et al., 2021), formal education is often not designed in a need-supportive way (Ryan and Deci, 2017). Oral exams constitute one of the strongest social-evaluative stressors (Zeidner, 1998) in education that may have a high impact (e.g., thesis defense) but often lack adequate examiner support (Buchwald and Schwarzer, 2003). Oral exams comprise time pressure, little feedback or feedback only as a final grade, and they are associated with uncertainty, lack of control, the necessity for quick reactions to exam questions and tasks, and high complexity, stemming from the direct interaction between examinee and examiner (Roick and Ringeisen, 2017; Ringeisen et al., 2019). This lack of “built-in” basic need support can result in a negative perception of the situation, thus in lower need satisfaction, higher stress, and lower achievement (Reeve and Tseng, 2011; Oberauer et al., 2016; Yu et al., 2018).

However, despite the general set-up, the interactive nature of oral exams offers the opportunity for examiners to support their examinees’ basic needs during the exam through their behavior. For example, an examiner could let the students choose the starting topic of a presentation, supporting their *need for autonomy*, i.e., students’ feeling of willingness, interest, or value in their actions. When examiners give informational feedback, they support students’ *need for competence*, i.e., the universal need to feel effective. When examiners behave in a caring, friendly, empathetic, and respectful way (Buchwald, 2002; Buchwald and Schwarzer, 2003), they support their students’ *need for relatedness*, i.e., the need to belong to, be involved with and cared for by others (Ryan and Deci, 2017, 2020). This basic need support could reduce stress responses, eventually improving student performance and achievement (Reeve and Tseng, 2011). Accordingly, investigating the impact of need support on well-being and achievement by means of an examiner’s behavior during oral exams offers an opportunity to improve formal education in a need-supportive, motivating, and healthy way (Ryan and Deci, 2017).

It is conceivable that not only perceived basic need support but also the individual’s basic need strength is essential for stress and achievement in oral exams (e.g., Vansteenkiste et al., 2020). For example, a student with a strong need for relatedness might try more than a student with a weak need for relatedness to engage in an emotionally responsive interaction with the examiner and, therefore, perceive more relatedness support. In oral exams, interindividual basic need differences might result in different basic need configurations in combination with need-supportive examiner behavior.

More detailed knowledge about occurring basic need strength and satisfaction configurations could help support a heterogeneous student body, consisting of different subgroups, to live up to their potential in the stressful event of an oral exam. This typological perspective has recently gained interest (Ryan and Deci, 2020) and is particularly useful in the investigation of the basic psychological needs in the context of oral exams. Person-centered approaches allow for an inclusion of the possibility that a sample includes “multiple subpopulations characterized by different sets of parameters” (Morin et al., 2016, p. 8), enabling an investigation of how many different classes can be found within data and how these classes differ from each other, also regarding different outcome variables and their development (Laursen and Hoff, 2006).

In the present case, the person-centered approach enabled an identification of subgroups with distinct configurations of basic psychological need intensity and support intensity that may be congruent or incongruent. In the congruent case, for example, a person has a high level of need strength as well as a high level of perceived need satisfaction. In the incongruent case, a person has, for example, a low level of need strength but at the same time a high level of perceived need satisfaction, which would mean over-satisfaction of the needs for that person. This differentiation is important because the three basic psychological needs are usually correlated and occur together naturally, and because each need is characterized by a specific intensity that may vary interpersonally (Ryan and Deci, 2017). Moreover, person-centered approaches are well-suited to address differences in group-specific patterns of the development of stress-related outcome variables (Laursen and Hoff, 2006) such as emotions, cortisol, or subjective stress perception, or performance outcomes such as grades, in association with the basic psychological needs (Vansteenkiste and Ryan, 2013). In this matter, prior research has found the configuration of the basic needs to have distinct associations with affect and well-being (e.g., Tóth-Király et al., 2020; Santana-Monagas and Núñez, 2022).

Accordingly, the current study investigated whether there are naturally occurring profiles based on the examinee’s basic need strength and perceived need support in oral exams, which has not been covered by empirical studies yet. In a second step, we analyzed whether the displayed profiles differed in stress responses and achievement to understand which intensity constellations of need strength and need support are associated with which stress- and performance-related outcomes. If there were specifically vulnerable or beneficial configurations, examiners might modulate their support behaviors according to the need strength of the subgroups or examinees might be screened for their need strength so that groups of varying need strength could be assigned to the most suitable examiner under consideration of their response to these need constellations. As such, examiners could help examinees live up to their possibilities by reducing stress through basic need support. It could enable new options to prepare students for and organize oral exams.

### Students’ basic psychological needs and need-supportive teacher behavior

Self-determination theory (SDT; Ryan and Deci, 2017) differentiates qualitatively distinct types of motivation. The most autonomous quality is intrinsic motivation. Intrinsically motivated behavior is performed for the sake of itself, e.g., out of curiosity or interest. Extrinsically motivated behavior is conducted for a purpose that is separable form the behavior. It can be integrated, identified, introjected, or externally regulated, i.e., more or less accepted and integrated into the self. The types of motivation stand on a continuum of relative autonomy, from controlled lower-quality to autonomous higher-quality motivation (Ryan and Deci, 2000). An individual’s environment, e.g., their teacher, can facilitate the emergence of high-quality motivation through basic need support: When students *perceive* that their teacher supports their needs, it can result in basic need satisfaction, subsequently promoting more autonomous motivation types like intrinsic motivation. For example, perceived need support is positively related to emotional well-being at the level of traits and daily fluctuations (Reis et al., 2000). A lack of perceived basic need support or even need frustration, on the other hand, promotes stress (Vansteenkiste and Ryan, 2013).

In education, students who perceive need support from their teachers are more prone to develop high-quality motivation, greater engagement, and better achievement (Ryan and Deci, 2020). For instance, perceived autonomy support predicted experienced interest in the classroom and could even attenuate a general decrease in students’ school motivation (Gillet et al., 2012). Moreover, the three basic needs are interrelated (Ryan and Deci, 2017). For example, autonomy support is positively linked to relatedness support. A teacher supporting autonomy by considering the student’s perspective might also be more responsive to relational concerns (Ryan and Deci, 2020). A greater sense of relatedness is further connected to a better relationship between student and teacher, fostering integration and, therefore, autonomous types of motivation, commitment, effort, satisfaction, engagement, achievement, and intellectual development (see overview by Hagenauer and Volet, 2014).

Investigating the role of perceived need support in oral exams, it seems necessary to also consider students’ basic need strengths, i.e., the relative salience or importance of the basic needs for the individual (Chen et al., 2015; Ryan and Deci, 2017). For example, it is conceivable that individuals with a strong need for autonomy might feel more stressed because of the exam-inherent time pressure and restrictions than those with a weak need for autonomy. These examples highlight the claim for “universality without uniformity” (Vansteenkiste et al., 2020, p. 17) in basic need research. While basic need satisfaction is of universal importance, basic need strength is acknowledged as a contributing factor with more subtle effects. Empirically, there are inconclusive findings regarding perceived need support, need strength, and their associations to (impaired) well-being. Research reported a minor albeit significant impact of need strength on the relation between need satisfaction and well-or ill-being that might be context-or situation-specific (Van Assche et al., 2018; Vansteenkiste et al., 2020). Accordingly, need strength might be associated with the relation between perceived need support and well-being in oral exams (Ryan and Deci, 2017). Therefore, research should consider both perceived basic need support in conjunction with basic need strength when investigating exam-related stress responses and achievement.

### Basic need support, stress, and oral exams

Stress is an organismic reaction to stressors like exams. Responses can be cognitive, e.g., lowered concentration, affective, e.g., increased subjective stress levels, and physiological, e.g., the reactivity of the hypothalamic–pituitary–adrenal (HPA) axis (Reeve and Tseng, 2011), as indexed by changes in acute cortisol concentrations. Higher perceived stress is generally associated with acutely higher cortisol levels, particularly in social contexts (Adam, 2012). In exams, characteristic changes in stress-related responses are expected across its stages, reflecting that uncertainty about the contents and the performance gradually decreases over time (Folkman and Lazarus, 1985; Carver and Scheier, 1994): Uncertainty and stress-related responses should be greatest during the *anticipatory stage* shortly before the exam, drop throughout the exam until the *waiting stage* commences once the exam is completed yet the grades are still unannounced. Afterward, stress responses should further decrease during the *outcome stage* once students have received feedback on their performance. These patterns are primarily confirmed for threat-related emotions such as anxiety, loss-related emotions such as anger or disappointment, and endocrinological responses such as saliva cortisol concentrations (Folkman and Lazarus, 1985; Carver and Scheier, 1994; Ringeisen and Buchwald, 2010; Bermeitinger et al., 2018; Ringeisen et al., 2019; Graham et al., 2022). However, some studies also found still elevated cortisol levels after the completion of oral exams (e.g., Preuß et al., 2010). Inverse patterns with gradual increases in intensity could be observed for challenge-related emotions such as hope and gain-related emotions such as relief (Folkman and Lazarus, 1985; Carver and Scheier, 1994; Ringeisen and Buchwald, 2010; Bermeitinger et al., 2018). Consequentially, reducing stress should be an important objective for examiners: Stress may be counterproductive during exams, and a steeper decline of stress-related symptoms may be associated with better performance (Ringeisen et al., 2019).

From a basic need perspective, stress responses might result from an overall lack of perceived need support during exams (Ryan and Deci, 2017; Campbell et al., 2018). The link of perceived need support to health and well-being and of need thwarting to ill-being (Vansteenkiste and Ryan, 2013) can be explained by stress: Need satisfaction in response to a positive event (e.g., perceived need support) is associated with anterior insula-based subjective feelings and their integration with reward processing in the striatum (Reeve and Lee, 2019), the brain’s reward center (Delgado, 2007; Haber, 2011). Striatum activity is linked to the adaptive regulation of the HPA axis, which is responsible for cortisol output (Heller et al., 2013). In short, basic need satisfaction by means of perceived need support and need frustration by thwarting are associated with activity in the body’s reward system, which influences subjective-affective stress responses accompanied by changes in acute cortisol secretion.

The influence of perceived need support on stress through need satisfaction has been corroborated in academic achievement contexts. Overall, teachers’ global basic need support negatively predicted stress levels in college students (Gilbert et al., 2021). Regarding interpersonal events like oral exams, autonomy-supportive teaching attenuated cortisol reactivity in students (Reeve and Tseng, 2011), while a lack of basic need satisfaction functioned as a stressor and resulted in worse daily functioning and poorer sleep quality and quantity during an exam period (Campbell et al., 2018). Basic need satisfaction, therefore, may influence learners’ stress responses. These findings provide important implications for oral exams: An examiner could influence the students’ subjective and endocrinological stress responses during exams indirectly through basic need support to help them live up to their full performance potential and minimize stress-related deterioration in achievement, for example, due to impaired retrieval of learned information (Reeve and Tseng, 2011; Oberauer et al., 2016; Yu et al., 2018; Ringeisen et al., 2019).

#### Typological analysis of basic needs

In oral exams, differences between need strength and the degree to which the desired need is met or missed can be perceived by students as various levels of need support, which should have corresponding effects on stress-related reactions during oral exams. Therefore, investigating the three basic needs of students concurrently and in conjunction with the corresponding need-supportive behaviors of examiners is helpful. However, basic need profiles in oral exams have not been explored yet. Such investigations may be realized using a typological, *person-centered* perspective, which groups individuals into profiles, allowing conclusions regarding individuals’ motivational profiles as a whole (Wang et al., 2017). In our case, configurations of *both* need strength *and* perceived need support for all three basic needs should be considered to create a holistic picture of need-related profiles in the oral exam context. Typological approaches include both cluster and latent class analysis. The latter illustrates combinations of motivational characteristics as they occur naturally. While the categorization of individuals using cluster analysis produces different results depending on the cluster method, latent class analysis groups people into relatively homogenous subgroups using a model-based method yielding more reliable results (Geiser, 2011; Fan et al., 2019). Therefore, latent class analysis was the chosen method in the current research.

Motivational research, including SDT research, has recently increased the usage of the typological, *person-centered* approach (Vansteenkiste et al., 2009; Ryan and Deci, 2020), underpinning and extending prior research (Vansteenkiste et al., 2009; Hayenga and Corpus, 2010), complementing the variable-centered perspective that is usually taken (Wormington and Linnenbrink-Garcia, 2017). New groupings of students according to motivational profiles (Martinent and Decret, 2015) or different distributions within profiles (Kusurkar et al., 2013) could implicate that different groups of students need different types of support provided by the teacher. So far, the person-centered perspective has focused mainly on configurations of intrinsic and extrinsic types of motivation in academic settings, not on the underlying basic needs. For example, Wang et al. (2017); Kusurkar et al. (2013); Hayenga and Corpus (2010); Baars and Wijnia (2018) investigated profiles of secondary and university students’ intrinsic and extrinsic motivation. Haerens et al. (2018) considered teaching styles and examined autonomy support and control. They all reported four profiles that basically differentiated between better and worse motivational quality, where intrinsic motivation was always associated with higher-quality profiles.

Therefore, identifying need-related classes of students may help researchers and lecturers to understand and foster the nature of their students’ motivation in oral exams (Ratelle et al., 2007). Specifically, latent class analysis enables the examination of class-specific changes in stress-related outcome variables (Laursen and Hoff, 2006; Tóth-Király et al., 2020; Santana-Monagas and Núñez, 2022) such as emotions, cortisol, or subjective stress perception, or performance outcomes such as grades, in association with the basic psychological needs (Vansteenkiste and Ryan, 2013). Considering that rising physio-affective stress may predict worse performance, for example, due to impaired memory retrieval under intensifying arousal, it seems likely that students’ membership to distinct groups based on configurations of need strength and need support could have indirect effects on exam performance through different associations with physio-affective stress-related variables (Reeve and Tseng, 2011; Oberauer et al., 2016; Yu et al., 2018; Ringeisen et al., 2019).

### Current study

The present study investigated students’ basic needs and responsive support behaviors of examiners in oral exams. Using latent class analyses, we examined whether there were groups of students that varied in their naturally occurring profiles across the strength and the perceived support of the three basic needs. Moreover, we investigated whether the expected examinee profiles differed in their stress response, indicated by subjective-affective and endocrinological changes, and their exam achievement, indicated by their achieved grade. To control for unwanted variability in variables that should not be affected by the exam procedures, we checked whether the profiles differed regarding other exam- and person-related control variables. We considered three hypotheses that guided our empirical investigation:

- *H1*: Aligned with the findings of prior research (e.g., Hayenga and Corpus, 2010; Kusurkar et al., 2013; Wang et al., 2017; Baars and Wijnia, 2018; Tóth-Király et al., 2020; Santana-Monagas and Núñez, 2022), we expected to find different profiles regarding basic psychological need strength and perceived need support. Because the basic psychological needs are correlated (Ryan and Deci, 2017) and both need satisfaction and need strength can be distinguished as contrastive pairs with low versus high levels (Vansteenkiste et al., 2009; Hayenga and Corpus, 2010), we expected to find four classes reflecting low need strength and low perceived support, low need strength and high perceived support, high need strength and high perceived support, and high need strength and low perceived support.
- *H2*: The support of the basic psychological needs is generally related to lower stress responses (Vansteenkiste and Ryan, 2013; Ryan and Deci, 2017, 2020; Campbell et al., 2018; Reeve and Lee, 2019). Thus, we expected higher-quality profiles (high perceived need support and high need strength levels, or higher perceived need support than need strength levels) to be associated with lower levels of loss-related emotions, lower perceived stress, lower cortisol, and higher levels of gain-related emotions compared to lower-quality profiles (low perceived need support and low need strength levels, or lower perceived need support than need strength levels).
- *H3*: As the support of the basic psychological needs is generally related to better performance (Ryan and Deci, 2017), we expected higher-quality profiles with more perceived need support to achieve better grades in the oral exam than lower-quality profiles with less perceived need support. As intensifying stress responses may impair performance (e.g., Oberauer et al., 2016; Ringeisen et al., 2019), we further examined whether class membership could have indirect effects on exam performance through different associations with physio-affective stress-related variables.

## Materials and methods

### Sample and procedure

Participants were *N* = 92 university students (*M* = 24.53 years old, *SD* = 3.07, *n* = 46 women) who attended a regular course on personality and social psychology at a German university, including a weekly lecture and accompanying tutorials. The response rate was 100%, i.e., all students of the course took the exam and participated in the study. Referencing the European Language Framework (Council of Europe, 2001), all participants were at least at C2-level in German (71.77% native German speakers), the highest global level ensuring full command of German for oral and written examinations. The non-native German participants reported Russian, Chinese, or Vietnamese as their mother tongue. All participants had lived in Germany for at least two years, with 74.6% of the participants raised in Germany. The study was designed according to the Declaration of Helsinki and approved by the University’s Ethics Commission. Participants gave written informed consent before data collection, knowing that participation was voluntary and their data would be treated with confidentiality.

The reported study was part of a larger research project on stress and coping in the context of oral exams (see Roick and Ringeisen, 2017; Bermeitinger et al., 2018). To complete the module mentioned above, students had to pass an oral exam, one of the strongest social evaluation stressors (Zeidner, 1998), that lasted about 30 min. The same examiner conducted all exams over the course of 14 days and was at that time blind to the hypotheses. The protocol of the oral exam was standardized, including the topics, question pool, wording of primary and follow-up questions, and feedback for all students. Standardization was important to ensure that class differences based on need strength and perceived need support represent interindividual response variability (*cf.* Herold et al., 2021). Specifically, the examiner supported the examinees’ basic needs moderately yet consistently (e.g., autonomy support: students could choose the topic they started the exam with; competence support: examiner provided verbal feedback during and after exam; relatedness support: friendly introduction and conduct of the exam). The co-examiner monitored need support consistently. In order to back up the sample for conducting analysis on interindividual response variability concerning need strength and perceived need support, we performed a power calculation for between factors ANOVA using G* Power 3.1 (Faul et al., 2007). Specifying the power test for a sample size of 92, four expected classes, and a power (1–β error probability) of 0.95, we got a critical *F* value of 2.708 and a required effect size of *f* = 0.442 (large effect) for class comparisons. As Table 1 shows, the classes differed in need strength and perceived need support with consistently large effects.

**Table 1:** Class comparisons of participant characteristics.

Variables	Class 1 Under–supported needs (need strength > need support)		Class 2 Over–supported needs (need strength < need support)		Class 3 Need strength and need support at low level		Class 4 Need strength and need support at high level		*p* ^a^	partial η^2^	post-hoc^b^
	*n*	*M* ± *SD*	*n*	*M* ± *SD*	*n*	*M* ± *SD*	*n*	*M* ± *SD*			
**Basic needs**											
Need strength autonomy (T1)	30	3.27 ± 0.32	25	3.11 ± 0.39	18	2.61 ± 0.25	19	3.43 ± 0.34	<0.001	0.423	2 < 4; 3 < 1,2,4
Need strength competence (T1)	30	3.21 ± 0.28	25	2.84 ± 0.29	18	2.64 ± 0.37	19	3.37 ± 0.33	<0.001	0.445	2 < 1,4; 3 < 1,4
Need strength relatedness (T1)	30	3.25 ± 0.36	25	2.77 ± 0.47	18	2.63 ± 0.38	19	3.46 ± 0.32	<0.001	0.417	2 < 1,4; 3 < 1,4
Need support autonomy (T3)	30	2.88 ± 0.27	25	3.33 ± 0.27	18	2.63 ± 0.29	19	3.78 ± 0.21	<0.001	0.717	3 < 1 < 2 < 4
Need support competence (T3)	30	2.81 ± 0.26	25	3.19 ± 0.22	18	2.56 ± 0.31	19	3.64 ± 0.28	<0.001	0.678	3 < 1 < 2 < 4
Need support relatedness (T3)	30	2.89 ± 0.25	25	3.53 ± 0.26	18	2.51 ± 0.46	19	3.87 ± 0.16	<0.001	0.753	3 < 1 < 2 < 4
**Controls**											
Age, years	30	24.70 ± 2.87	25	25.20 ± 3.69	18	23.50 ± 2.6	19	24.79 ± 3.38	0.373	0.035	
Sex (% female)	30	43.3	25	60.0	18	44.4	19	52.6	0.613		
Average awakening time	28	8.08 ± 1.28	24	8.18 ± 1.86	18	8.33 ± 1.03	19	8.11 ± 1.13	0.941	0.005	
Body mass index, kg/m^2^	30	22.68 ± 3.15	24	23.22 ± 4.16	18	23.72 ± 3.37	19	25.47 ± 4.84	0.103	0.068	
Importance of performance (T1)^c^	30	3.97 ± 0.93	25	3.68 ± 1.03	18	3.67 ± 0.77	19	4.05 ± 0.97	0.418	0.031	
Intensity of preparation (T1)^c^	30	3.10 ± 0.80	25	2.92 ± 0.95	18	3.11 ± 0.83	19	3.00 ± 1.05	0.870	0.008	
Expected performance (T1)^d^	30	2.47 ± 0.58	25	2.70 ± 0.75	18	2.60 ± 0.70	19	2.30 ± 0.69	0.242	0.046	
**Performance**											
Achieved grade (T4)^e^	30	2.59 ± 0.89	25	2.60 ± 0.80	18	3.13 ± 1.01	19	2.47 ± 0.91	0.113	0.065	

^a^All statistical comparisons performed via ANOVAs, except sex via χ^2^ -tests; ^b^Bonferroni correction; ^c^1 = “not a bit” … 5 = “extremely”; ^d^1 = “very good” (A) … 4 = “sufficient” (D); ^e^German grading system: lower values indicate better performance: 1 = “very good” (A) … 5 = “failed” (E).

**Figure 1:**
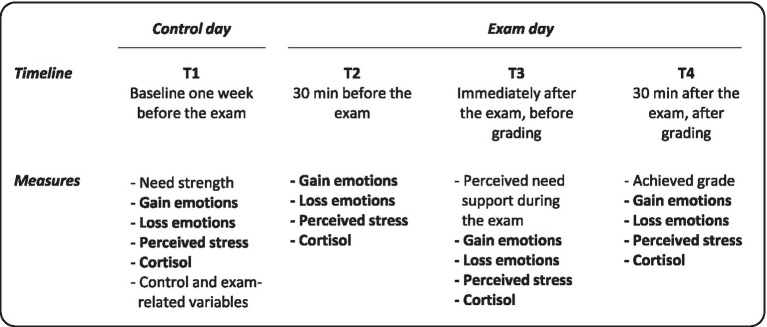
Assessment design and measures. Measures which were assessed longitudinally are printed in bold font.

The study comprised *four* measurement points: A control day one week before the exam (T1) and three points of measurement on the exam day itself (*cf.* Schoofs et al., 2008; Preuß et al., 2010), namely 30 min before the exam (T2), directly after the exam but before the announcement of the grade (T3), and about 30 min after the exam, after the announcement of the grade (T4) (Figure 1). Thereby, our design covered the temporal stages of an exam (Folkman and Lazarus, 1985), namely the *anticipatory stage*, the *waiting stage*, and the *outcome stage*. Initially, it was planned to have an additional measurement point right before the start of the exam to ensure intervals of 30 min between assessments on the exam day, which the Ethics Commission denied. Using paper-pencil-questionnaires, participants reported their basic need strength at T1, their perceived basic need support during the exam retrospectively at T3, and perceived stress and gain- and loss-related outcome emotions at all four measurement points. We followed the recommendations by Chan (2009) to use self-report data; hence, we operationalized all constructs strictly according to theory, used only scales validated for the respective language group, and randomized the order of measures.

Complementarily to subjective stress and emotions, cortisol levels were measured with salivary samples from T1 to T4. Cortisol levels do not only vary in response to the onset of a stressor (acute cortisol response) but also throughout the day (diurnal cortisol pattern) (Dickerson and Kemeny, 2004; Foley and Kirschbaum, 2010), with a peak cortisol concentration about 30 min after waking up and a subsequent continuous decrease (Hellhammer et al., 2009). All oral exams started at different times between 9 am and 4 pm for practical reasons. Therefore, we implemented an intraindividual control design to control for participants’ baseline cortisol concentrations and individual diurnal cortisol patterns. We asked for the participants’ awakening time and parallelized the timing of the cortisol assessment times at T1 and T2, also ensuring that the time lag between waking up and cortisol assessment at T1 and T2 was equal. Besides, we considered the unwanted effects of demographic variables, medical conditions, or long-term medication that may also influence cortisol concentrations. For example, being over- or underweight (body mass index BMI > 30 kg/m^2^ or < 17.0 kg/m^2^) can have confounding effects on cortisol concentrations (Foley and Kirschbaum, 2010).

In terms of control variables, we assessed person-related information such as age, sex, height and weight (to calculate the BMI), medical condition, the use of hormonally active long-term medication, and the average awakening time at T1. In addition, we assessed selected exam-related variables (the expected grade, the intensity of exam preparation, and the importance of performing well at T1; the achieved grade at T4).

### Instruments and measures

#### Need strength and perceived need support

Due to the specific German oral exam setting, we used validated scales in German that mirror the wording and subscale structure of the widely used instrument by Chen et al. (2015). Aligned with considerations on trait assessment in academic performance settings (Zeidner, 1998; Tibubos et al., 2019), we operationalized need strength as a situation-specific trait with reference to exam-related tasks or examiner behaviors. Specifically, we investigated autonomy and competence strength employing the two respective scales from Rakoczy et al. (2005), enriched by items by Katz and Cohen (2014). We measured social relatedness strength by adapting a questionnaire designed by Seidel (2012). Items were introduced with “It is important to me that …” and followed by, for example, “the examiner gives me hints so I can solve tasks by myself” (competence strength, nine items), “I have the opportunity to work through the topics autonomously with the guidance of the examiner” (autonomy strength, six items), and “the examiner gives me a sense of belonging” (relatedness strength, six items). Students indicated “rarely,” “sometimes,” “often,” or “very often” on a 4-point Likert scale.

To assess the extent to which students perceived the examiner to satisfy their needs during the exam, participants evaluated the examiner’s behavior at T3 in retrospect with the same set of items. However, we adapted the wording to past tense and the introductory phrase to “During the oral exam ….” Students indicated how often they perceived the respective aspect on a 4-point scale from “not at all” to “to a great deal.” For example, “It is important to me that I feel the examiner meets my needs and understands me” (social relatedness strength) became “During the oral exam, I felt that the examiner met my needs and understood me” (perceived social relatedness support), “It is important to me that I have the opportunity to deal with tasks or topics that interest me in more detail” (autonomy strength) became “During the oral exam, I could deal with tasks or topics that interested me in more detail” (perceived autonomy support), and “It is important to me that I get help when I cannot solve a task by myself” (competence strength) became “During the oral exam, the examiner helped me when I could not answer a task/question” (perceived competence support). Except for autonomy strength (*α* = 0.64), reliability of the need strength and perceived need support scales was acceptable, ranging from *α* = 0.77 to *α* = 0.88.

#### Gain- and loss-related outcome emotions

With three adjectives each, we assessed the level at which participants perceived themselves as satisfied, happy, and relieved, mirroring gain-related emotions, and as angry, disappointed, and guilty, mirroring loss-related emotions, at each measurement point (Carver and Scheier, 1994; Ringeisen and Buchwald, 2010). Cronbach’s alphas (*α* = 0.77, 0.60, 0.61, and 0.78 for gain-related emotions and *α* = 0.54, 0.79, 0.75, and 0.85 for loss-related emotions from T1 to T4, respectively) were similar to or even slightly above those in the cited studies. Participants were asked, “Please specify to which extent the following descriptions apply to you when you think about the oral exam now,” followed by a list of the above-named adjectives. They indicated their answer on a five-point scale from “not at all” (1) to “extremely” (5).

#### Subjective stress experience

We assessed the participants’ subjective stress experience using the Visual Analog Scale (VAS, Luria, 1975), yielding high to very high correlations with longer scales measuring stress-related affect (Gallagher et al., 2002). Students marked on a continuous line of exactly 10 cm from “no stress” on the left-hand side to “maximum stress” on the right-hand side how stressed they perceived themselves regarding the oral exam.

#### Cortisol assessment

Cortisol levels were measured in saliva. Saliva was collected with a shortened straw into polypropylene microtubes (SafeSeal, Sarstedt). Samples were then frozen at −20°C, thawed, vortexed, and centrifuged for 15 min at 2500 Å ~ g (Function Line 400R, Heraeus) twice. Before analysis, the supernatant was transferred in duplicate into a pre-coated microwell plate. Cortisol concentration was quantified by an immunoassay kit (IBL, Hamburg, Germany). Two samples had to be excluded due to blood contamination caused by gum bleeding or injuries in the participant’s mouth, affecting subsequent measurements (Westermann et al., 2004). A 96-well ELISA reader (Thermo Fisher) was used for saliva analyses and intra-assay coefficients of variance below 5% and inter-assay coefficients below 11% were reported by a professional laboratory.

#### Exam-related variables

We measured the expected and achieved exam grade with one single open-response item, as recommended by the German Association to Foster Educational Research (Rakoczy et al., 2005; adapted from Carver and Scheier, 1994) because research has shown that single-item measures may be used effectively in similar education settings (e.g., Leung and Xu, 2013). Students answered the questions “What grade do you think you will achieve in the oral exam?” (expected grade; T1) and “Which grade did you achieve in the exam?” (achieved grade; T4). Participants expected to pass the exam with good to satisfactory grades (*M* = 2.52, SD = 0.68) and achieved well satisfactory grades, on average (*M* = 2.67, SD = 0.91). Please note that lower numbers signify better grades because the German university grading system ranges from 1 (“A”) as the best grade to 5 (“E”) as the worst grade. Participants also indicated the importance of their performance at T1 (Rakoczy et al., 2005). Participants answered the question “How important is it for you to do well in the oral exam” on a scale from “not at all” (1) to “extremely” (5). It was important to them to perform well (*M* = 3.85, SD = 0.94). To assess the intensity of preparation, we asked participants the question “How well-prepared do you feel regarding the upcoming exam?” (Ringeisen, 2008), which they answered on a 5-point scale from “not at all” (1) to “extremely” (5) (*M* = 3.03, SD = 0.91).

#### Person-related control variables

Participants indicated their age and sex as well as height and weight. The BMI could be calculated from the latter two variables. Besides, participants reported any medical condition “Do you suffer from any illness/health impairment (e.g., a cold, diabetes)? If yes, which?” and the intake of medication “Do you currently take any medication (e.g., aspirin, hormonal contraceptives)? If yes, which?.” To control for awakening times, participants reported the awakening time on the control day at T1 and their average awakening time during the week between the control day and the exam day by answering the question “At what time do you usually get up (*ca.* average time across the last 7 days).”

### Statistical analysis

For (repeated measures) ANOVAs, correlations, and regressions, we used IBM® SPSS® Statistics Version 27, and Mplus Version 8.5 (Muthén and Muthén, 1998/2017) for latent class analyses. Before analysis, the data set was cleaned. Because of possible confounding effects on cortisol concentrations, the saliva of students with a severe medical condition and of those using any hormonally active long-term medication or reporting being over−/underweight (BMI > 30 kg/m2 or < 17.0 kg/m2) was not analyzed (Foley and Kirschbaum, 2010). Consequently, the final data set contained saliva samples from 80 students and questionnaire data from all 92 students.

To assess interindividual differences in needs and perceived need support, we used a person-centered approach and analyzed data *via* latent class analysis (LCA; Geiser, 2011; Dziak et al., 2014). In general, LCA identifies homogenous subgroups in a sample using continuous variables (Marsh et al., 2009; Geiser, 2011). The classification into groups or different classes was made based on the mean scales for each of the need variables. To determine the most adequate number of need classes, different solutions were tested and compared based on the following indices: Loglikelihood (LL), Akaike Information Criteria (AIC), Bayesian Information Criteria (BIC), sample-size adjusted BIC (ssaBIC), Mendell–Rubin-Likelihood-Ratio-Tests (VLMRT, aLMRT), Bootstrap LRT, and the mean class membership probabilities. For AIC, BIC, and ssaBIC, lower values generally indicate a better model fit when comparing models with different numbers of classes (Geiser, 2011; Dziak et al., 2014). However, Nylund-Gibson and Choi (2018) stated that it is not uncommon that the BIC, such as other information criteria, continue to decrease for each additional class added. In this case, according to the authors, the point of diminishing returns should be examined, the so-called “elbow.” LMRT and Bootstrap LRT provide values directly comparing the calculated model with a defined number of classes with a model that contains one class less (Nylund et al., 2007; Geiser, 2011). Concerning mean class membership probabilities, Weller et al. (2020) state that values between 0.8 and 0.9 are acceptable. All measures are therefore indicators of relative model fit. Another criterion is the so-called entropy, where a value of > 0.8 indicates an acceptable classification. Weller et al. (2020) stated that the entropy values of the class solutions should be reported and investigated but should not be used to determine the final class solution. Last but not least, Marsh et al. (2009) recommended that a profile should not be made of less than 5% of the sample size.

Subsequently, we compared the identified latent classes on the stress-related state variables (emotions, subjective stress perception, cortisol concentrations), person-related control variables, and exam-related control variables. First, we calculated Pearson-Correlations for measures of need variables and mean changes in gain-related emotions, loss emotions, perceived stress, and cortisol values. Second, to compare change patterns in stress-related state variables between classes, we conducted repeated-measures ANOVAs for gain-related emotions, loss emotions, perceived stress, and cortisol concentration, with TIME of measurement as the repeated within-subjects factor and CLASS as the between-subjects factor. Mean differences between groups for each point of measurement were tested *via* ANOVAs. Third, we compared the classes regarding baseline-corrected, relative changes in stress-related state variables. Considering interindividual variability in baseline values, we subtracted the values at T1 from the following values at T2, T3, and T4, as recommended by Roberts et al. (2004) and Kärner et al. (2018) and tested for corrected relative mean changes (averaged sums of baseline-corrected data) *via* ANOVAs.

To examine the direct effects of class membership and indirect effects of physio-affective stress-related variables on performance, we conducted a mediation analysis with a multi-categorical independent variable following the general description of Hayes and Preacher (2014) (the statistical computations were carried out using Mplus version 8.5; Muthén and Muthén, 1998/2017). For this purpose, the mean changes over time of the variables gain-related emotions, loss-related, perceived stress and cortisol levels were used as mediators and separate models were calculated for each mediator variable.

## Results

### Associations between the study variables

Screening the Pearson correlations yielded several significant associations (see Table 2). We found medium to strong positive associations between the three facets of need strength (0.446 ≤ *r* ≤ 0.737) and strong associations between the three facets of need support (0.760 ≤ *r* ≤ 0.795). Facets of need strength reflected significant weak to medium positive associations with facets of need support (0.162 ≤ *r* ≤ 0.415). Mean increases in gain-related emotions were associated with greater need strength autonomy (*r* = 0.305), and greater perceived need support regarding autonomy (*r* = 0.359), relatedness (*r* = 0.381), and competence (*r* = 0.222). Mean increases in cortisol values were related to lower values in need strength autonomy (*r* = −0.357), need strength competence (*r* = −0.264), perceived need support autonomy (*r* = −0.278), and need support relatedness (*r* = −0.267). The relative changes in the two stress-related measures (perceived stress and cortisol measures) correlate negatively (*r* = −0.154) but not significantly with each other.^1^ Concerning performance, higher need strength competence (*r* = −0.241) and more gain-related emotions experienced during the test (*r* = −0.379) are significantly associated with better test results.

**Table 2:** Pearson correlations between the study variables.

*Variables*		1	2	3	4	5	6	7	8	9	10
1.	Need strength autonomy	–									
2.	Need strength competence	0.661***	–								
3.	Need strength relatedness	0.446***	0.737***	–							
4.	Need support autonomy	0.415***	0.303**	0.223*	–						
5.	Need support relatedness	0.358***	0.324**	0.284**	0.795***	–					
6.	Need support competence	0.331***	0.247*	0.162	0.760***	0.769***	–				
7.	Gain emotions^a^	0.305**	0.094	0.095	0.359***	0.381***	0.222	–			
8.	Loss emotions^a^	−0.008	0.028	0.062	0.039	0.085	0.041	−0.053	–		
9.	Perceived stress^a^	0.097	0.097	−0.030	−0.082	−0.047	−0.060	−0.150	−0.066	–	
10.	Cortisol values^a^	−0.357**	−0.264*	−0.143	−0.278*	−0.267*	−0.185	−0.157	−0.093	−0.154	–
11.	Achieved grade^b^	−0.202	−0.241*	−0.176	−0.155	−0.190	−0.124	−0.379	0.172	−0.066	0.082

73 ≤ *n* ≤ 92; ***p ≤ 0.001; **p ≤ 0.01, *p ≤ 0.05; ^a^Mean changes over time; ^b^German grading system: lower values indicate better performance: 1 = “very good” (A) … 5 = “failed” (E).

### Identification and characterization of classes

Table 3 contains the model fit information for latent class models with 1 to 6 classes. Weighing statistical criteria for cluster identification, we selected the four-classes solution for subsequent analysis, which appeared to fit the data best, although the fit indices provided a somewhat mixed indication of the best-fitting number of classes.^2^ The information criteria (AIC, BIC, ssaBIC) favored the four-classes solution reflecting a diminishing decrement in information criteria values for each added class (the so-called “elbow”). For the four-classes solution, the entropy value was 0.860, indicating an acceptable classification (*cf.* Weller et al., 2020), and all cell frequencies were above the recommended 5% of the total sample (*cf.* Marsh et al., 2009). In order to back up the four-classes solution, we conducted a split-half cross-validation (*cf.* Fu and Perry, 2020). We divided the sample randomly into two subsamples (A and B) and calculated the four-classes solution for each of the two subsamples. The solution obtained with the two subsamples and the solution obtained with the full sample matched each other accurately (Pearson *χ*^2^ = 184.60, *p* < 0.001; Cramer’s *V* = 0.818, *p* < 0.001; Contingency Coefficient = 0.817, *p* < 0.001). The mean differences in the variables need strength and perceived need support between the classes-solution generated on the total sample and the classes-solution generated on the subsamples were consistently not significant. On this basis, we performed cross-validation. Each participant from subsample B was assigned to the class from subsample A whose variable values were closest to the centers of the classes from subsample A. Comparing the class assignment on the basis of class centers and the classes determined *via* class analysis for subsample B indicated accuracy (Pearson *χ*^2^ = 44.68, *p* < 0.001; Cramer’s *V* = 0.569, *p* < 0.001; Contingency Coefficient = 0.702, *p* < 0.001). Even given the relatively small sample size, the results indicate adequate stability of the four-classes solution.

**Table 3:** Model fit information for latent class models with 1–6 classes.

		No. of classes					
		1	2	3	4	5	6
Cell frequencies per class	1	92	51	33	30	9	5
	2		41	9	25	22	24
	3			50	18	19	24
	4				19	25	3
	5					17	21
	6						15
**Model fit information**							
No. of free parameters		12	19	26	33	40	47
LL		−370.006	−293.622	−264.948	−236.487	−220.193	−207.583
AIC		764.011	625.244	581.896	538.974	520.386	509.166
BIC		794.273	673.158	647.462	622.193	621.258	627.690
ssaBIC		756.394	613.184	565.392	518.027	494.996	479.332
**Diminishing returns**			1 → 2	2 → 3	3 → 4	4 → 5	5 → 6
Diff. AIC			−138.77	−43.35	−42.92	−18.59	−11.22
Diff. BIC			−121.12	−25.70	−25.27	−0.93	6.43
Diff. ssaBIC			−143.21	−47.79	−47.37	−23.03	−15.66
Entropy		NA^a^	0.865	0.892	0.860	0.884	0.901
VLMRT		NA^a^	0.014	0.224	0.225	0.344	0.611
aLMRT		NA^a^	0.016	0.233	0.233	0.352	0.615
PBLRT		NA^a^	<0.001	<0.001	<0.001	<0.001	<0.001

LL, Loglikelihood; AIC, Akaike’s Information Criterion; BIC, Bayesian Information Criterion; ssaBIC, Sample-size adjusted Bayesian Information Criterion; VLMRT, Vuong–Lo–Mendell–Rubin Likelihood-Ratio Test (value of *p*); aLMRT, Lo–Mendell–Rubin adjusted Likelihood-Ratio Test (value of *p*); PBLRT, Parametric Bootstrap-Likelihood-Ratio Test (value of *p*), ^a^not available for the one-class model.

In addition to the model fit criteria and the split-half cross-validation, the mean class membership probabilities also indicate an acceptable four-classes solution because all group-related average probabilities exceed the threshold of 0.9 (Table 4).

**Table 4:** Mean class membership probabilities.

Membership probabilities	Participants of Class 1		Participants of Class 2		Participants of Class 3		Participants of Class 4	
	*M*	SE(M)	*M*	SE(M)	*M*	SE(M)	*M*	SE(M)
MP for Class 1	0.903	0.025	0.048	0.016	0.047	0.019	0.001	0.001
MP for Class 2	0.042	0.021	0.910	0.028	0.003	0.002	0.045	0.021
MP for Class 3	0.041	0.014	0.002	0.001	0.957	0.015	0.000	0.000
MP for Class 4	0.000	0.000	0.050	0.027	0.000	0.000	0.950	0.027

MP, Membership probabilities.

To characterize the class configurations, we compared the four classes with regard to need strength and need support. Autonomy strength (*p* < 0.0001; η_p_^2^ = 0.423), competence strength (*p* < 0.0001; η_p_^2^ = 0.445), and relatedness strength (*p* < 0.0001; η_p_^2^ = 0.417) as well as autonomy satisfaction (*p* < 0.0001; η_p_^2^ = 0.717), competence satisfaction (*p* < 0.0001; η_p_^2^ = 0.678), and relatedness satisfaction (*p* < 0.0001; η_p_^2^ = 0.753) differed significantly between the four classes. Post-hoc Bonferroni tests revealed that autonomy strength was significantly lower in Class 2 (*M* = 3.11, SD = 0.39) than in Class 4 (*M* = 3.43, SD = 0.34) and in Class 3 (*M* = 2.61, SD = 0.25) than in Class 1 (*M* = 3.27, SD = 0.32), Class 2 (*M* = 3.11, SD = 0.39), and Class 4 (*M* = 3.43, SD = 0.34). Competence strength was significantly lower in Class 2 (*M* = 2.84, SD = 0.29) than in Class 1 (*M* = 3.21, SD = 0.28) and 4 (*M* = 3.37, SD = 0.33) and in Class 3 (*M* = 2.64, SD = 0.37) than in Class 1 (*M* = 3.21, SD = 0.28) and Class 4 (*M* = 3.37, SD = 0.33). The same counted for relatedness strength (Class 1: *M* = 3.25, SD = 0.36; Class 2: *M* = 2.77, SD = 0.47; Class 3: *M* = 2.63, SD = 0.38; Class 4: *M* = 3.46, SD = 0.32). All need satisfactions were lower in Class 3 (autonomy: *M* = 2.63, SD = 0.29; competence: *M* = 2.56, SD = 0.31; relatedness: *M* = 2.51, SD = 0.46) than in Class 1 (autonomy: *M* = 2.88, SD = 0.27; competence: *M* = 2.81, SD = 0.26; relatedness: *M* = 2.89, SD = 0.25) than in Class 2 (autonomy: *M* = 3.33, SD = 0.27; competence: *M* = 3.19, SD = 0.22; relatedness: *M* = 3.53, SD = 0.26) than in Class 4 (autonomy: *M* = 3.78, SD = 0.21; competence: *M* = 3.64, SD = 0.28; relatedness: *M* = 3.87, SD = 0.16).

**Figure 2:**
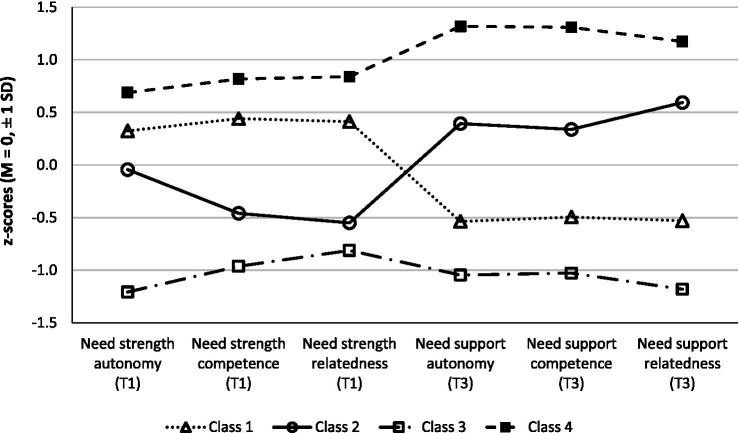
Class profiles concerning need strength (T1) and perceived need support (T3).

In summary, the four classes can be characterized in relation to each other as follows (Table 1; Figure 2):

- *Class 1 (high/low)*. Participants with *above-average* scores on need strength (autonomy, competence, relatedness) and *below-average* scores on perceived need support (autonomy, competence, relatedness)
- *Class 2 (low/high)*. Participants with *below-average* scores on need strength (competence, relatedness), an *average score* on need strength autonomy, and *above-average* scores on perceived need support (autonomy, competence, relatedness).
- *Class 3 (low/low). Consistently below-average* values for both need strength and perceived need support.
- *Class 4 (high/high)*. *Consistently above-average* values for both need strength and perceived need support.

The four classes did not differ significantly concerning the person-related control variables (age, sex, average awakening time, and body mass index) and the exam-related control variables (subjective importance of exam performance, intensity of preparation for the exam, and expected performance). Thus, the classes were comparable concerning the control variables, which had been measured before the exam at T1. Unexpectedly, the achieved grade at T4 also did not differ between classes. Only Class 3 and Class 4 displayed the expected tendency descriptively (*M* = 3.13 for Class 3, *M* = 2.60 for Class 2, *M* = 2.59 for Class 1, and *M* = 2.47 for Class 4, note that lower scores indicate better performance in the German grading system) (Table 1).

### Class comparisons regarding needs, emotions, and stress

#### Comparison of baseline values

Table 5 shows the results of group comparisons of baseline values at T1. The four classes did not differ significantly concerning gain-related emotions, perceived stress, and cortisol values, all *p* > 0.05. However, Class 3 showed significantly higher values of loss emotions at T1 (*M* = 2.61, SD = 0.87) than Class 2 (*M* = 1.83, *SD* = 0.59; medium effect, *p* = 0.007, η_p_^2^ = 0.128).

**Table 5:** Class comparisons of baseline values at T1.

Variables	Class 1 Under-supported needs (need strength > need support)		Class 2 Over-supported needs (need strength < need support)		Class 3 Need strength and need support at low level		Class 4 Need strength and need support at high level		*p* ^a^	partial η^2^	post–hoc^b^
	*n*	*M* ± *SD*	*n*	*M* ± *SD*	*n*	*M* ± *SD*	*n*	*M* ± SD			
**Baseline Values at T1**											
Gain emotions	30	2.57 ± 0.99	25	2.12 ± 0.69	18	2.78 ± 0.58	19	2.53 ± 0.79	0.055	0.082	
Loss emotions	30	2.11 ± 0.72	25	1.83 ± 0.59	18	2.61 ± 0.87	19	1.98 ± 0.76	0.007	0.128	3 > 2
Perceived stress (VAS)	30	5.09 ± 3.12	24	4.40 ± 2.92	17	6.11 ± 3.11	19	4.36 ± 2.91	0.258	0.046	
Cortisol, nmol/L	27	14.7 ± 8.88	18	21.45 ± 14.51	17	13.08 ± 8.57	14	15.98 ± 6.84	0.081	0.089	

^a^All statistical comparisons performed via ANOVA. ^b^Bonferroni correction.

#### Class differences in mean changes over time and repeated measures ANOVAs

Table 6 shows the results of group comparisons of mean changes over time. Participants in Class 2 showed significantly higher values in the mean change (indicating increases over time) in gain-related emotions (*M* = 1.20, SD = 0.67) compared to participants in Class 3 (*M* = 0.29, SD = 0.77, *p* = 0.003, η_p_^2^ = 0.145). Furthermore, participants from Class 3 showed significantly higher mean changes (indicating increases over time) in cortisol values (*M* = 21.35, SD = 15.45) compared to participants from Classes 2 (*M* = 2.44, SD = 13.52) and 4 (*M* = 6.30, SD = 12.09, *p = 0.*002, η_p_^2^ = 0.188).

**Table 6:** Class comparisons of mean changes.

Variables	Class 1 Under-supported needs (need strength > need support)		Class 2 Over-supported needs (need strength < need support)		Class 3 Need strength and need support at low level		Class 4 Need strength and need support at high level		*p* ^a^	partial η^2^	post–hoc^b^
	*n*	*M* ± SD	*n*	*M* ± SD	*n*	*M* ± SD	*n*	*M* ± SD			
*Mean changes* ^c^											
Gain emotions	30	0.67 ± 0.96	25	1.20 ± 0.67	18	0.29 ± 0.77	19	0.96 ± 0.72	0.003	0.145	3 < 2
Loss emotions	30	0.21 ± 0.76	25	0.18 ± 0.95	18	−0.03 ± 0.71	19	0.19 ± 1.04	0.797	0.011	
Perceived stress (VAS)	30	−1.17 ± 2.42	24	−0.85 ± 2.68	17	−1.30 ± 2.01	19	−1.42 ± 3.03	0.895	0.007	
Cortisol, nmol/L	27	9.98 ± 13.98	18	2.55 ± 13.52	16	21.35 ± 15.45	14	6.30 ± 13.09	0.002	0.188	3 > 2,4

^a^All statistical comparisons performed via ANOVA. ^b^Bonferroni correction.^c^Mean baseline-corrected values from T2 to T4.

**Figure 3:**
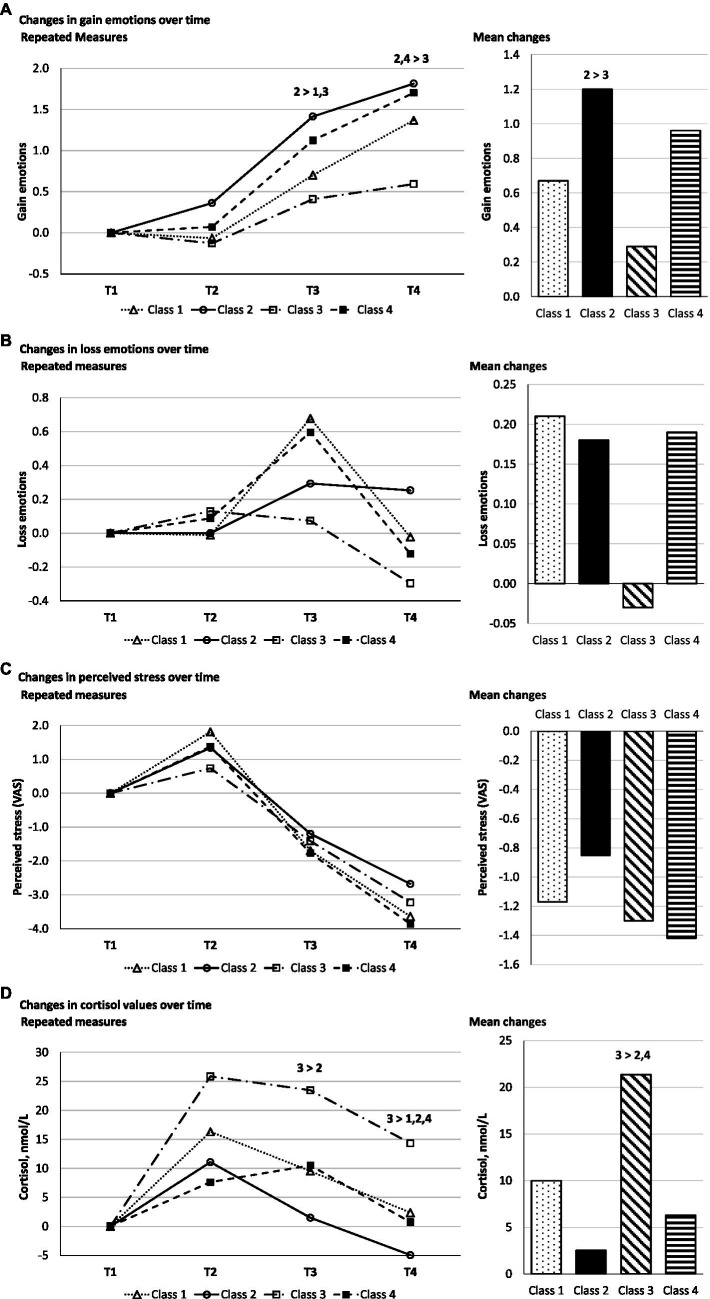
Gain–and loss–related emotion, perceived stress, and cortisol (mean) changes over time.

Repeated measures ANOVAs displayed significant *main effects for TIME* for all four stress-related state variables, indicating a significant increase of gain-related emotions (*p* < 0.001, η_p_^2^ = 0.461), decreases for perceived stress (*p* < 0.001, η_p_^2^ = 0.602), and cortisol concentration (*p* < 0.001, η_p_^2^ = 0.267), after a peak at T2, and a peak for loss emotions (*p* < 0.001, η_p_^2^ = 0.135) at T3, followed by a decrease (Table 7; Figure 3). In accordance with the results described for Table 1, we found significant *main effects of CLASS* for gain-related emotions (*p* < 0.0001, η_p_^2^ = 0.145) and cortisol concentration (*p* < 0.003, η_p_^2^ = 0.199) but not for loss emotions (*p* = 0.797, η_p_^2^ = 0.011) and perceived stress (*p* = 0.895, η_p_^2^ = 0.007). At T3, Class 2 displayed significantly higher gain-related emotions (*M* = 1.41, SD = 0.81) than Class 1 (*M* = 0.70, SD = 1.03) and Class 3 (*M* = 0.41, SD = 0.99). At T4, Class 3 (*M* = 0.59, SD = 1.36) displayed significantly lower gain-related emotions than Class 2 (*M* = 1.81, SD = 1.07) and Class 4 (*M* = 1.70, SD = 1.04). At T3, Class 3 displayed significantly higher cortisol values (*M* = 23.46, SD = 24.03) than Class 2 (*M* = 0.17, SD = 12.74). At T4, Class 3 displayed significantly lower cortisol levels (*M* = 13.38, SD = 14.43) than Class 1 (*M* = 2.07, SD = 10.50), Class 2 (*M* = −5.07, SD = 11.08), and Class 4 (*M* = 0.75, SD = 12.10).^3^ A significant *interaction effect between TIME and CLASS* was found for loss emotions (Table 7; Figure 3B). Classes 1, 3, and 4 showed a decrease in loss emotions from T3, whereas class 2 showed a slight increase (*p* < 0.011, η_p_^2^ = 0.091).

**Table 7:** Repeated measures (repeated-measures ANOVAs).

Variable	Effect	df	*F*	Greenhouse–Geisser value of *p*	Partial η^2^
Gain emotions	TIME	1.802	75.367	<0.001	0.461
	CLASS	3	4.985	0.003	0.145
	TIME × CLASS	5.406	1.698	0.133	0.055
Loss emotions	TIME	1.840	13.785	<0.001	0.135
	CLASS	3	0.339	0.797	0.011
	TIME × CLASS	5.521	2.950	0.011	0.091
Perceived stress	TIME	1.987	130.067	<0.001	0.602
	CLASS	3	0.201	0.895	0.007
	TIME × CLASS	5.960	1.097	0.366	0.037
Cortisol	TIME	1.483	22.924	<0.001	0.267
	CLASS	3	5.213	0.003	0.199
	TIME × CLASS	4.449	1.153	0.337	0.052

#### Mediation analysis with class as multicategorical independent variable

**Figure 4:**
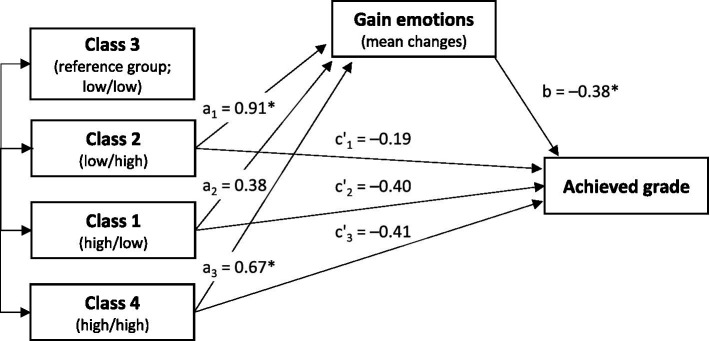
Mediation analysis with Class as multicategorical independent variable. Reference group: Class 3. Achieved grade according to the German grading system (lower values indicate better performance): 1 = “very good” (A) … 5 = “failed” (E). *p < 0.05. Relative indirect effects: Class 2 (a_1_b = −0.35*), Class 1 (a_2_b = −0.14), Class 4 (a_3_b = −0.26*). Unstandardized values.

Using Class 3 (low/low) as a reference group, the results of the mediation analyses indicated a significant indirect effect in the magnitude of −0.35 (*p* < 0.05) for Class 2 (low/high) and in the magnitude of −0.26 (*p* < 0.05) for Class 4 (high/high) on performance mediated by gain-related emotions: Individuals in Class 2 and Class 4 (the high-quality classes) experienced more gain-related emotions, which were in turn significantly associated with better grades (Figure 4). Using Class 1 (high/low) as a reference group within the mediation analysis, only the indirect effect for Class 2 (−0.201, *p* = 0.041) remained significant, but not the indirect effect for Class 4 (−0.113, *p* = 0.227). To summarize, the pattern of evidence indicates that Class 2 benefitted most in terms of gains associated with emotions that were perceived as positive. In the other models calculated (loss-related emotions, stress experience and cortisol levels as mediators), there were no significant mediation effects on performance for any of the considered classes.

## Discussion

### Summary and interpretation of findings in light of our hypotheses

The current study investigated basic need strength and perceived basic need support classes in a real-life oral exam. The person-centered perspective enables theoretical and practical implications for basic need support in stressful social-evaluative contexts in formal education. We identified four significantly distinct basic need configurations, supporting Hypothesis 1. Class 1 comprised students whose experienced basic need support and strength were incongruent, because their indicated levels of need strength were higher than their levels of perceived need support. Class 2 comprised students whose basic need strength and perceived support were also incongruent, with higher perceived need satisfaction compared to their indicated need strengths. Need strength and perceived need support were congruent in Class 3, with consistently below-average values, and in Class 4, with consistently above-average values for both need strength and perceived need support.

Classes 2 and 4 displayed higher perceived need satisfaction and more adaptive developments in gain-related emotions and cortisol, indicating higher quality. Classes 1 and 3 reflected lower-quality classes with less perceived need support and less beneficial gain-related emotions. In correspondence with the assumption of self-determination theory that the basic psychological needs are universal but the effects of their satisfaction may also be associated with the respective need strength (Ryan and Deci, 2017), basic need strength seemed to further differentiate the higher-quality (Classes 2 and 4) and lower-quality (Classes 1 and 3) classes by the perceived under-/over-support of the initially indicated need strength levels, i.e., when need strength and perceived need support levels were incongruent. Over-support seems to enhance gain-related emotion development and could buffer heightening cortisol concentrations, while under-support could have opposite effects (Figure 3).

The emerging classes underline the importance of integrating the typological approach into motivational research in education and conformed with prior research focusing on different types of intrinsic and extrinsic motivation (e.g., Vansteenkiste et al., 2009; Ryan and Deci, 2017; Baars and Wijnia, 2018). The basic needs occur together naturally, and the support of one need may simultaneously support the other needs (Chen et al., 2015; Ryan and Deci, 2017), which is reflected in the correlations between the perceived support levels of the three needs, and the strength levels of the three needs in the present study.

Basic need support is related to well-being (Ryan and Deci, 2017), so teachers’ basic need support may reduce stress in college students (Gilbert et al., 2021). We assumed that higher-quality classes with more perceived need support could be associated with more beneficial stress-related outcomes than lower-quality classes (Hypothesis 2). Our data partly supported this assumption. Combinations of both low need strength and low perceived need support levels displayed the lowest gain-related emotions and highest cortisol concentrations compared to the higher-quality classes, particularly the class with over-supported needs. This is in line with the physiological links of basic need support to well-being: When the basic needs are satisfied as a response to need support (Reeve and Tseng, 2011), the striatum, i.e., the brain’s reward center (Delgado, 2007; Haber, 2011), is activated. Being rewarded feels good, hence the higher levels of perceived gain-related emotions and lower cortisol segregation of the HPA axis (Heller et al., 2013) in participants higher-quality compared to the lower-quality basic need configurations. However, while other authors reported associations between perceived stress and basic need satisfaction (e.g., Weinstein et al., 2016; Campbell et al., 2018) and although stress and uncertainty about the contents and performance gradually decreased from before until after the exam (Folkman and Lazarus, 1985; Carver and Scheier, 1994), there were no significant differences between the classes in our sample concerning changes in *perceived* stress and loss-related emotions.

We assume that different response areas (e.g., subjective stress perception, emotions, or performance) might be triggered differently by basic need support because of how basic need support works in the human body. Stress perception and cortisol levels could differ because HPA activation of cortisol needs 15–20 min after the onset of the stressor, while emotional responses happen immediately (Dickerson and Kemeny, 2004). The significant time-lagged correlations between perceived stress (T1) and cortisol values at T2 and T4 could be explained by anticipation in terms of a presumption of the stressful oral exam (see footnote 1). Further, perceived stress was questioned rather nonspecifically, while emotions were indicated more specifically with concrete indicators for emotional experience. Still, Cronbach’s α of gain- and loss-related emotions were rather low for some measurement points, indicating that positive emotion ratings varied intra-individually before and after the exam. In contrast, negative emotion ratings varied a week before the exam but converged on the exam day. This pattern suggests that students’ experiences of positive emotions might have been complex, precise, and multi-faceted during the exam, signifying high emotional granularity for positive emotions, whereas emotional granularity for negative emotions could have been low. Previous research found positive emotional granularity to be associated with characteristic psychophysiological responses, greater psychological resilience, and more effective coping in the face of social-evaluative stress (Tugade et al., 2004). When cortisol is released in response to a perceived (lack of) reward, as indicated by perceived emotions, it might explain why more cortisol was released when low need strength was combined with little perceived need support, while increases in gain-related emotions were smaller compared to classes with strong perceived need support.

Literature shows that basic need support is related to better performance (Ryan and Deci, 2017), so we expected participants in higher-quality classes with high perceived need support to perform better in the oral exam than participants in lower-quality classes (Hypothesis 3). The classes did not differ significantly in the achieved grades after the exam, contrasting other research (e.g., Hayenga and Corpus, 2010; Kusurkar et al., 2013; Baars and Wijnia, 2018). Our findings suggest differentiated relations. At a correlational level, we found greater competence strength and more intense gain-related emotions to be associated with better achieved grades, which corroborates previous findings on the role of competence-related positive emotions for performance during need-supportive oral examinations (Pekrun, 2006). Notably, Classes 2 and 4 reported more intense gain-related emotions than Class 3, and these differences to gain-related emotions in Class 3 were positively associated with differences in achievement. Particularly Class 2, where perceived need satisfaction exceeded the indicated need strength, appeared to benefit most with regard to gains associated with positive emotions. Meeting or even over-supporting the indicated strength of the needs through need support could result in more intensive gain-related emotions, which are linked to higher achievement in exams. This finding highlights the necessity to constructively consider learners’ emotional states and needs in didactic efforts and to understand and acknowledge learning-related emotions as a constitutive element in the acquisition of knowledge (Sembill, 1992).

Our findings could imply that participants in lower-quality classes, particularly those with under-supported needs (Class 3), could show a tendency towards basic need frustration (e.g., Vansteenkiste and Ryan, 2013). Their indicated need strength levels met or were higher than the already low perceived need support levels, while participants in higher-quality classes with higher perceived need support might have felt need satisfaction during the exam because their high perceived need support levels met or exceeded the indicated need strength levels. Thus, high perceived need support appears to be beneficial for higher-quality basic need classes to occur, especially under consideration of an individual’s need strength. More vulnerable students with low basic need strength, who might not be as actively claiming need support from their teachers as students with high need strengths, could profit from need-supportive teachers. Therefore, the findings underline that, while the basic psychological needs and the associations of their satisfaction are universal, the individual’s need strength can alter the effects of the experienced support (Ryan and Deci, 2017) and could help examiners choose the most adequate behavior during oral exams.

Overall, the existence of different subgroups of examinees regarding their basic psychological need configurations and their distinct relations to stress-related state variables and achievement implies that it is relevant to consider these configurations in the preparation of examinees and examiners for oral exams.

### Limitations and strengths of the study

Despite the study’s strengths, some methodological limitations should be acknowledged. First, future studies might replicate the current investigation with larger samples that might reveal smaller but significant effects that we could not detect in this case (Field, 2009). However, multiple-measurement studies assessing both affective and endocrinological stress responses are complex and often limited to sample sizes between about 50 and 100 participants for practical reasons (e.g., Zeidner, 1998; Campbell and Ehlert, 2012; Graham et al., 2022). Second, we used self-report measures for students’ anticipated grades, personal relevance, emotions, stress, and perceived behavior. We were interested in students’ respective *perceptions* because basic need satisfaction depends on *perceived* need support. Perceived and actual relatedness support are interrelated (Reeve and Jang, 2006; Haerens et al., 2013), but we cannot distinguish whether perceived basic need support stemmed from behavioral differences in need support *actually provided by the teacher* or students’ perception. We followed Chan’s (2009) advice to address construct validity, interpretation of correlations, social desirability response, and value of data collected from other sources as typical challenges of self-report data. Moreover, we utilized cortisol as an additional measure for stress. Third, although the co-examiner checked for standardized support behavior during the exam, future research might profit from recordings of the exam situations to rate the examiner’s support behavior, which was not possible due to data security in the current study. Fourth, our study focused on oral exams, so our findings are not generalizable to other exam forms like written exams without consideration of the respective specific characteristics of each method of examination (Tibubos et al., 2019). Fifth, future research could assess perceived need frustration configurations more directly and in various contexts. Need frustration could explain tendencies in perceived stress even more (Ryan and Deci, 2017; Schürmann and Quaiser-Pohl, 2022). It is conceivable that the present study did not report differences in loss-related emotions and stress perception between the higher- and the lower-quality classes, also in association with achievement, because we focused on perceived need support and, therefore, need satisfaction. Future research could consider need frustration as an option to explain group differences in negative affective state variables such as loss-related emotions or perceived stress due to the asymmetric relationship between need satisfaction and frustration (Vansteenkiste and Ryan, 2013; Santana-Monagas and Núñez, 2022). Moreover, the degree of change in the stress-related variables could make a difference. Perhaps there is a certain threshold that has to be met for effects to occur. Sixth, although the latent class analysis was the best approach to investigate whether there were subgroups that differed in their basic need configurations and associated stress-related state variables and achievement in an oral exam to better prepare both examinees and examiners for this situation, further research is needed as our findings might not be representative of other samples. Seventh, while difficult to realize in the context of real-life oral exams, future research could profit from bigger sample sizes when using LCA. Eighth, it can be assumed that the link between emotions and academic performance might go both ways. Emotions may not only foster academic achievement but could also follow from it. Thus, at T4, the announcement of the grade might influence students’ emotions, as well.

Beyond these limitations, the study has distinct strengths compared to prior research. First, we assessed the data in a real-life situation, including real students in real exams with real consequences. This supports both external and ecological validity of oral exams as one of the most threatening social contexts for students (Zeidner, 1998; Dickerson and Kemeny, 2004). Second, we measured cortisol levels in a longitudinal design to track intraindividual cortisol changes and reveal class differences between the lower and higher-quality classes in the context of the typical development of cortisol levels in exams (Ringeisen et al., 2019). Third, to our knowledge, this study is the first to examine the relation of basic need configurations, stress symptomatology, and performance in oral exams from a typological perspective (e.g., Ryan and Deci, 2020). Knowing that students differ in motivational configurations, examiners could adapt their behavior to minimize possible negative influences of stress on academic performance and thereby better focus on students’ actual intellectual ability. Moreover, the study shows that basic need support works even in very stressful, formal, standardized settings, offering important theoretical and practical implications.

### Implications for instructional research and teaching practice

The findings underline the importance of integrating the typological perspective to research on basic needs in education. The present study adds to the research on configurations of different types of intrinsic and extrinsic motivation (e.g., Ratelle et al., 2007; Vansteenkiste et al., 2009; Baars and Wijnia, 2018) by focusing on the basic needs (e.g., Haerens et al., 2018). We identified four classes of basic need configurations, two higher-quality classes with high perceived need support and two lower-quality classes with lower perceived need support, in an oral exam.

It is an implication for practice that need support constitutes an efficient and harmless option to ease *all* students’ perceptions of oral exams as a stressful event. Thus, examiners could support their students’ basic needs in exam situations. Need support might result in higher perceived need satisfaction (Reeve and Jang, 2006; Haerens et al., 2013), which could, eventually, promote more autonomous types of motivation. Examiners may support their students’ needs by slightly altering their behavior. For example, they could shift from disregarding students’ feelings to welcoming them and being attentive to their basic needs by acknowledging their perception of the situation. Therefore, it is crucial to educate practitioners about motivation theory (Schürmann et al., 2021) and, more specifically, basic need support.

## Conclusion

The current study closes the research gap concerning the relation between the basic needs, need support, stress symptomatology, and performance during oral exams. We found four classes that differed regarding stress symptomatology. The lowest-quality class with the lowest need strength and perceived need support displayed the highest cortisol levels and lowest gain-related emotions, while the higher-quality classes displayed reversed tendencies. Meeting or even over-supporting the needs appeared as most beneficial because particularly high levels of gain-related emotions mediated the positive relation of these classes to achievement. Overall, the findings suggest that the more supportive the examiner’s behavior is perceived by the examinees, particularly exceeding their need strength, the greater their perceived need support and resulting need satisfaction and the greater the beneficial effects on the examinees’ emotional and physiological stress reactions during the exam could be. Thus, future research should include the typological perspective on the basic needs, extend its areas of interest to other contexts, and further investigate the predictive power of basic need support for emotions, perceived stress, cortisol, and performance.

## Data availability statement

The raw data supporting the conclusions of this article will be made available by the authors, without undue reservation.

## Ethics statement

The studies involving human participants were reviewed and approved by University of Merseburg. The patients/participants provided their written informed consent to participate in this study.

## Author contributions

LS contributed to writing—original draft, review, editing, and verification. TK contributed to methodology, formal analysis, writing—original draft, review, editing, and visualization. TR contributed to conceptualization, verification, investigation, resources, writing—review, editing, and supervision. All authors contributed to the article and approved the submitted version.

## Conflict of interest

The authors declare that the research was conducted in the absence of any commercial or financial relationships that could be construed as a potential conflict of interest.

## Publisher’s note

All claims expressed in this article are solely those of the authors and do not necessarily represent those of their affiliated organizations, or those of the publisher, the editors and the reviewers. Any product that may be evaluated in this article, or claim that may be made by its manufacturer, is not guaranteed or endorsed by the publisher.

## Footnotes

^1^Approximately 75% of existing studies do not report a link between subjective stress and cortisol levels, an effect that strengthens when single measures are aggregated and mean changes are correlated (Campbell and Ehlert, 2012). To additionally examine the validity of the two stress-related measures, the time-related raw values were correlated with each other. Reporting only the significant correlations, results show that perceived stress (T1) is significantly positively correlated with cortisol values at T2 (*r* = 0.234, *p* = 0.048) and T4 (*r* = 0.240, *p* = 0.041) and that perceived stress at T2 is significantly positively correlated with cortisol values at T4 (*r* = 0.266, *p* = 0.021). These correlations show time-lagged associations between perceived stress and subsequent cortisol responses during the examination period, corroborating time-lagged associations around *r* = 0.20 (Campbell and Ehlert, 2012).

^2^We also discussed a two-class solution, consisting of a Class 1 with significantly lower need for autonomy strength at T1 compared to a Class 2 (medium effect), but no significant group differences for need for competence and need for relatedness. Compared to Class 1, participants of Class 2 displayed significantly higher levels of perceived autonomy, competence, and relatedness support at T3 (large effects). Class 1 of the two-class solution corresponded to Classes 1 and 3 of the four-classes solution, and Class 2 corresponded to Classes 2 and 4, respectively. Therefore, the two-class solution signifies a less fine-grained variant of the described four-classes solution.

^3^The respective calculations were conducted for each point of measurement. The values may deviate slightly from the values in Figure 3D, because the values in Figure 3D are based on the values of the repeated-measurements ANOVA that utilizes listwise deletion.
